# Metabolic Engineering and Regulation of Diol Biosynthesis from Renewable Biomass in *Escherichia coli*

**DOI:** 10.3390/biom12050715

**Published:** 2022-05-18

**Authors:** Tong Wu, Yumei Liu, Jinsheng Liu, Zhenya Chen, Yi-Xin Huo

**Affiliations:** Key Laboratory of Molecular Medicine and Biotherapy, School of Life Science, Beijing Institute of Technology, No. 5 South Zhongguancun Street, Haidian District, Beijing 100081, China; 3120205672@bit.edu.cn (T.W.); 3120191366@bit.edu.cn (Y.L.); 3120201437@bit.edu.cn (J.L.); huoyixin@bit.edu.cn (Y.-X.H.)

**Keywords:** diols, metabolic engineering, carbon sources, regulation, *Escherichia coli*

## Abstract

As bulk chemicals, diols have wide applications in many fields, such as clothing, biofuels, food, surfactant and cosmetics. The traditional chemical synthesis of diols consumes numerous non-renewable energy resources and leads to environmental pollution. Green biosynthesis has emerged as an alternative method to produce diols. *Escherichia coli* as an ideal microbial factory has been engineered to biosynthesize diols from carbon sources. Here, we comprehensively summarized the biosynthetic pathways of diols from renewable biomass in *E. coli* and discussed the metabolic-engineering strategies that could enhance the production of diols, including the optimization of biosynthetic pathways, improvement of cofactor supplementation, and reprogramming of the metabolic network. We then investigated the dynamic regulation by multiple control modules to balance the growth and production, so as to direct carbon sources for diol production. Finally, we proposed the challenges in the diol-biosynthesis process and suggested some potential methods to improve the diol-producing ability of the host.

## 1. Introduction

Fossil resources such as oil, petroleum and natural gas are the primary fuels and important chemical raw materials [1]. However, non-renewable petroleum energy has not satisfied the needs of industrial development, leading to an increasingly prominent energy crisis. Furthermore, petroleum-based-chemical manufacturing could influence the economy, the environment, and even the global security [2,3,4]. As one of the renewable resources, diols could also meet the needs of growing global energy and reduce adverse environmental impacts. With the rapid development of molecular biology and synthetic biology, biosynthetic diols have gradually become the substitute for traditional fuels. Nowadays, diols such as ethylene glycol (EG), 1,2-propanediol (1,2-PDO), 1,3-propanediol (1,3-PDO), 2,3-butanediol (2,3-BDO) or 1,4-butanediol (1,4-BDO) are widely applied in various areas including antifreeze agents [5], polyurethanes [6], surfactants [7] and cosmetics [8]. For instance, the global market for EG is about 15 million tons and valued at more than $20 billion per year [9]. As a kind of chemical intermediate, 1,4-BDO is utilized in various industries such as clothing and electronic products, or as a basic raw material for producing PBT fiber [10]. The global market value of 1,4-BDO has reached $6.19 billion, and it is expected to reach a record level of $12.6 billion by 2025 [11].

To date, diol production mainly relies on chemical synthesis from non-renewable energy resources [9]. For example, 1,2-PDO is chemically synthesized by the hydration of propylene or glycolysis at high pressure and temperature. However, a racemic mixture is obtained, and the titer of the target product is fairly low [12]. 1,4-BDO is produced mainly from fossil materials such as acetylene, butane or propylene, and the production process is accompanied by greenhouse gas emissions. Thus, the biosynthesis of diols by designing and establishing metabolic pathways is urgently needed to be developed.

As an ideal microbial cell factory, *E. coli* has a clear genetic background and a mature gene-manipulation toolbox even though various microorganisms such as *Klebsiella pneumoniae*, *Bacillus licheniformis* or *Bacillus amyloliquefaciens* could naturally produce diols. Metabolic engineering is an efficient tool to enhance the ability of the chassis to synthesize diols, because it could endow microorganisms with the heterologous synthesis of compounds and improve the production of different compounds. To produce biofuels or other valuable chemicals, numerous biosynthetic pathways have been designed and constructed in different chassis. Here, this review summarizes the construction of biosynthetic pathways of representative diols, and analyzes the current metabolic-engineering strategies and their effects on optimizing diol production. Moreover, we consider the unsolved difficulties in promoting the production and suggest many potential methods to further optimize and enhance the productivity of diols in *E. coli*.

## 2. Biosynthetic Pathways of Diols in *E. coli*

**Figure 1:**
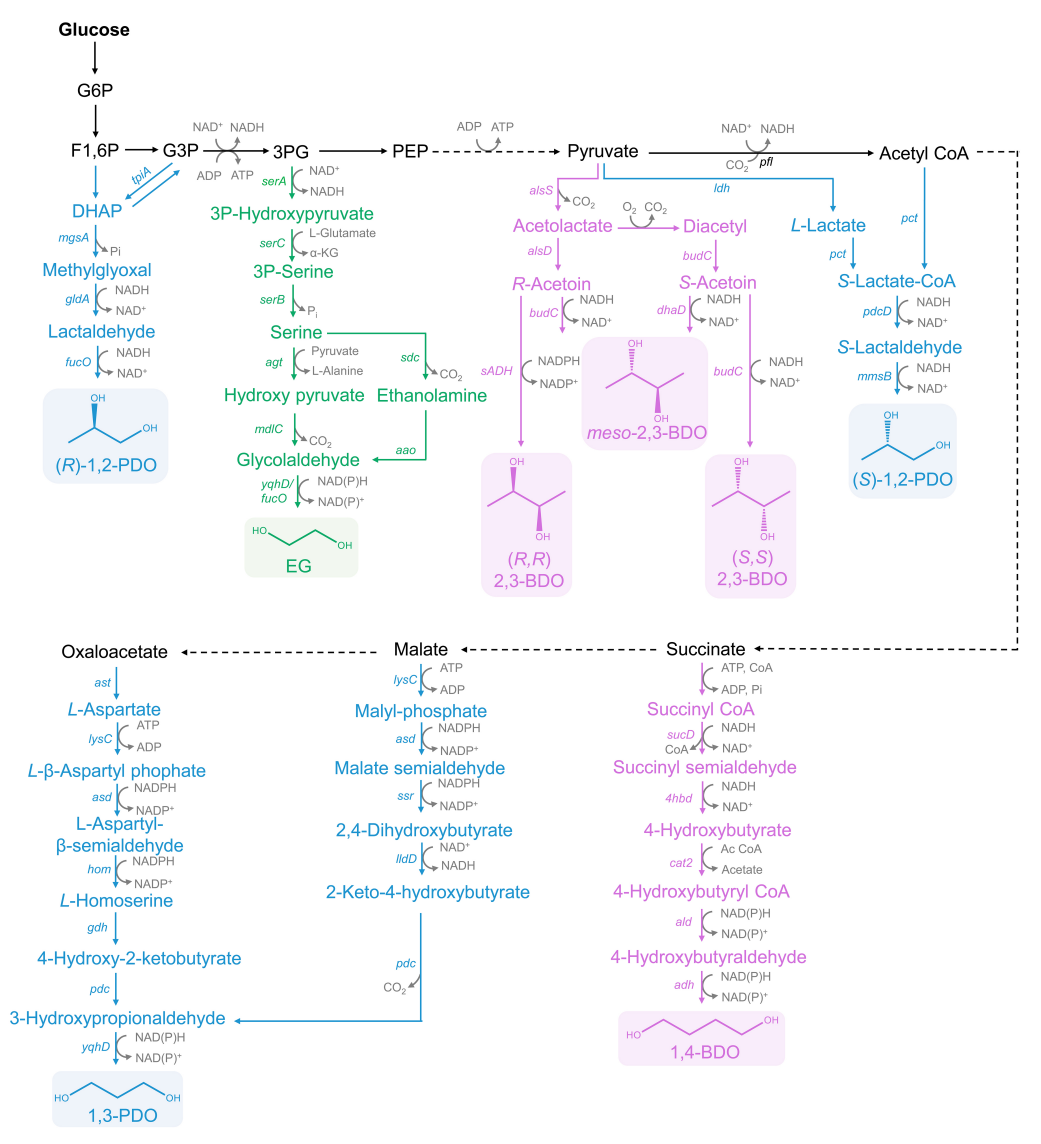
Biosynthetic pathways of diols from glucose in *E. coli. mgsA*: methylglyoxal synthase; *gldA*: glycerol dehydrogenase; *fucO*: alcohol dehydrogenase; *tpiA*: triose-phosphate isomerase; *serA*: phosphoglycerate dehydrogenase; *serC*: phosphohydroxythreonine aminotransferase; *serB*: phosphoserine phosphatase; *agt*: ecarboxylase; *yqhD*: alcohol dehydrogenase; *alsS*: acetolactate synthase; *alsD*: α-acetolactate decarboxylase; *budC*: BDO dehydrogenase; *dhaD*: glycerol dehydrogenase; *sADH*: BDO dehydrogenase; *ldh*: *L*-lactate dehydrogenase; *pfl*: pyruvate formatelyase; *pct*: propionate CoA-transferase; *pdcD*: aldehyde dehydrogenase; *mmsB*: alcohol dehydrogenase; *sucD*: succinate semialdehyde dehydrogenase; *4hbd*: 4-hydroxybutyrate dehydrogenase; *cat2*: 4-hydroxybutyryl-CoA transferase; *ald*: 4-hydroxybutyryl-CoA transferase; *adh*: alcohol dehydrogenase; *lysC*: malate kinase; *asd*: malate semialdehyde dehydrogenase; *ssr*: malate semialdehyde reductase; *lldD*: *L*-lactate dehydrogenase; *pdc*: OHB decarboxylase; *ast*: aspartate aminotransferase; *hom*: homoserine dehydrogenase; *gdh*: glutamate dehydrogenase.

Glucose, a cheap sugar with an extensive value in use, is the preferred carbon source for the biosynthetic production of diols. Glucose is gradually decomposed into a series of intermediates with the catalysis of enzymes by glycolysis and the TCA cycle. The generated intermediates in each step are the important precursors for the production of different diols. The biosynthetic pathways of the representative diols based on the order of the intermediate formation and the length of carbon chains are summarized in Figure 1.

3-Phosphoglycerate (3PG) is the origin of EG production. Under the successive catalysis of SerA, SerC and SerB, serine is generated from 3PG. Serine could be catalyzed into hydroxy-pyruvate by serine-glyoxylate aminotransferase AGT, or ethanolamine by serine decarboxylase SDC. These two intermediates could be transformed to glycolaldehyde via the catalysis of α-keto acid decarboxylase MDLC, or ethanolamine oxidase AAO. In the end, glycolaldehyde is oxidized into EG under the expression of YqhD or FucO. The whole process produces one molecule of NADH and consumes one molecule of NAD(P)H.

For 1,2-PDO biosynthesis, dihydroxyacetone phosphate (DHAP) is converted from fructose-1,6-phosphate (F1,6P). Especially, DHAP and glyceraldehyde 3-Phosphate (G3P) could be mutually transformed. DHAP is catalyzed into methylglyoxal (MG) by MG synthase. After two reduction reactions, (*R*)-1,2-PDO is generated. In contrast, for the synthesis of (*S*)-1,2-PDO by direct fermentation from glucose, the biosynthetic design is based on the proven pathway from organic acids to diols [13]. *S*-Lactate-CoA could be generated from pyruvate or acetyl CoA. (*S*)-1,2-PDO is biosynthesized under the catalysis of aldehyde dehydrogenase PdcD and alcohol dehydrogenase MmsB [5].

Both malate and oxaloacetate can be gradually converted to 1,3-PDO. Malate can be gradually transformed into 2-keto-4-hydroxybutyrate (OHB) under the catalysis of malate kinase LysC, the malate semialdehyde dehydrogenase Asd, malate semialdehyde reductase Ssr and DHB dehydrogenase LldD, respectively. At the same time, *L*-homoserine is obtained from oxaloacetate that the pathway naturally exists in *E. coli*. Subsequently, *L*-homoserine is converted into 3-hydroxypropionaldehyde (3-HPA) by glutamate dehydrogenase GDH and OHB decarboxylase PDC. Similarly, OHB can also be transformed into 3-HPA by PDC. Finally, 1,3-PDO is synthesized from 3-HPA by an aldehyde reductase YqhD.

Commonly, 2,3-BDO contains three isomeric forms: *meso*-2,3-BDO, (*S*, *S*)-2,3-BDO and (*R*, *R*)-2,3-BDO. As for 2,3-BDO biosynthesis, three key enzymes are involved: α-acetolactate synthase ALS, α-acetolactate decarboxylase ALDC, and BDO dehydrogenase BDH, also known as acetoin reductase. In the first step, two molecules of pyruvate are transformed into one molecule of acetolactate via acetolactate synthase ALS, and then ALDC catalyzes acetolactate to *R*-acetoin. (*R*, *R*)-2,3-BDO or *meso*-2,3-BDO could be obtained by the catalysis of BDO dehydrogenase with the consumption of one molecule of NAD(P)H. For the formation of (*S*, *S*)-2,3-BDO, acetolactate is spontaneously catalyzed to diacetyl in the presence of oxygen, and diacetyl is further transformed to *S*-acetoin by diacetyl reductase DAR. (*S*, *S*)-2,3-BDO is generated by (*S*, *S*)-BDO dehydrogenase BDH. Additionally, as an intermediate, *S*-acetoin could be transformed to *meso*-2,3-BDO under the catalysis of glycerol dehydrogenase encoded by *dhaD*.

1,4-BDO is biosynthesized from succinate. Succinate is converted to succinyl-CoA by succinyl-CoA synthetase SCS. Next, succinyl-CoA is reduced to 4-hydroxybutyrate (4-HB) in two successive steps, which need the catalysis of CoA-dependent succinate semialdehyde dehydrogenase SucD and 4-hydroxybutyrate dehydrogenase 4HBd. Subsequently, 4-HB-CoA transferase catalyzes 4-HB into 4-HB CoA. 4-HB CoA is reduced to 4-hydroxybutyraldehyde by 4-HB-CoA reductase. Finally, 1,4-BDO is produced by alcohol dehydrogenase. This bio-pathway consumes one molecule of ATP and four molecules of NAD(P)H.

## 3. Metabolic-Engineering Strategies for Diol Biosynthesis

Currently, the biosynthetic pathways of diols from glucose have been realized in *E. coli* by combining the endogenous and exogenous pathways. However, the current yield and productivity cannot satisfy the demands of industrialization. Based on this urgent purpose, various metabolic-engineering strategies including optimization of the biosynthetic pathways of diols, optimization of cofactor supplementation, and reprogramming of the metabolic network have been designed and established. The concrete details are summarized in Table 1.

### 3.1. Optimization of Biosynthetic Pathways of Diols

#### 3.1.1. Redesign of the Biosynthetic Pathways

The pathways of 1,3-PDO and 1,4-PDO rely on the formation of *L*-homoserine and the supply of cofactor CoA, respectively. However, the deamination rate of *L*-homoserine and the amount of CoA in the host restrict the production of 1,3-PDO and 1,4-PDO. For this reason, Wang et al. redesigned the diol pathways based on the natural metabolic pathways of amino acids (aspartate or glutamate) in *E. coli*. The productive process was approximately divided into three parts: the synthesis of amino acids, the degradation of amino acids, and the formation of diols. A carboxylic-acid reductase Car, from *Mycobacterium marinum* was chosen to reduce noncognate ω-hydroxylic acids (ω-HAs). With the overexpression of Car and YqhD, ω-HAs were degraded into diols [26]. However, the titers of the diols were still at a low level that could not satisfy the commercialization benchmark. Later, Liu et al. established the bio-pathways of diverse diols combing oxidative and reductive formations of OH groups. By using enzyme-catalyzed substrates at a particular carbon site, diols could be biosynthesized from various amino acids [27].

Due to the existence of endogenous pathways, diols can be easily synthesized from amino acids. Hence, amino-acid overproducers are indispensable for diol production. Various tools for the screening and selection of amino-acid overproducers, such as an amino-acid-biosensing system based on rare-codon or codon-extension strategies have been established [28,29,30]. Zheng et al. developed a biosensor system that could respond to the concentration of intracellular amino acids by introducing rare codons into marker genes such as resistance genes. The existence of rare codons can inhibit the sequence-translation rate under the condition of amino-acid starvation, and this effect can be enhanced with the increase in the number of rare codons. By increasing the concentration of amino acids in the cells, the inhibiting effect can be alleviated to some extent. When the host produces amino acids with a high yield, the amino acids can be gradually accumulated in the cell to remove the inhibiting effect. The resistance genes can be further expressed and the host can be alive in a double-resistant-culture medium. Hence, these tools can be utilized to screen a host with a high production of diols by amino-acid overproducers.

#### 3.1.2. Knockout of the Competing Pathways

Besides the redesign of the synthetic pathways, the knockout of the competing pathways in the host is also crucial for the elevation of diol biosynthesis, which could affect the direction of the metabolic flux. Nowadays, bulk commercial chemicals need a “factory” in order to be overproduced from a common carbon resource [31]. As a non-native producer, although *E. coli* could produce diols via the exogenous pathways, there are still quite a few deficiencies such as a low efficiency of substrate conversion and poor chassis adaptation. Furthermore, the microbial production of diols is subject to carbon loading and end-product inhibition by diols and other end-products [32]. To avoid the influence of the above-mentioned effects on diol production, Kim et al. removed the competing pathways to ethanol and glycerol with the introduction of the 2,3-BDO biosynthetic pathway. The metabolic flux was successfully redirected to produce the target product [33]. Similarly, Jain et al. destroyed ethanoic-acid synthesis to make the carbon flux flow to production. In addition, the first committed step of ubiquinone biosynthesis was disrupted, which prevented the oxidation of NADH. By combining these two modifications, the yield of 1,2-PDO was dramatically enhanced from 0.2 g L^−1^ to 1.2 g L^−1^ [34]. In order to steadily produce (*S*)-1,2-PDO, Zhu et al. integrated the bio-pathway into the genome, and the strain was engineered to delete the genes responsible for undesired lactate utilization, which could avoid the generation of *D*-lactic acid and the additional consumption of glucose in other branches of metabolic pathways [5]. With the normalization of the knockout of the competing pathways for the optimization of compound biosynthesis, diverse gene-engineering techniques have been established, especially CRISPR/Cas9 and base editors that can inactivate the key genes in the competing pathways. CRISPR/Cas9 can break the double-stranded DNA to cause gene knockout through homologous recombination. Base editors require the fusion of a deaminase to the inactive Cas9 protein. Under the guidance of sgRNA and formation of R-loop, the fusion protein can directly edit the target site and catalyze the deamination of the base. Moreover, due to the fact that base editors do not rely on double-stranded breaks (DSBs) or HR (homologous recombination), it is more convenient to achieve efficient gene editing compared with CRISPR/Cas 9 [35]. Therefore, both of these methods could implement the destruction of the competing pathways.

#### 3.1.3. Selection of Key Enzymes for the Target Configurational Diols

Except for the optimization of the host, highly active enzymes are also critical for constructing efficient diol biosynthetic pathways. The modification and optimization of the enzymes could hoist the production capability of chassis and the yield of products. Because of the multiple stereo forms of some diols, their substrate specificity is considered for the selection of exogenous enzymes. As a diol with three isomers, the insufficient purity of 2,3-BDO would emerge. The enzyme that plays a decisive role in 2,3-BDO configuration is BDO dehydrogenase BDH. Consequently, Yan et al. chose three different BDHs to synthesize (*R*, *R*)-2,3-BDO. They are encoded by *bdhA* from *Bacillus subtilis*, *adh* from *Clostridium beijerinckii*, or *adh* from *Thermoanaerobium brockii*, respectively. All of the selected BDHs have strict stereospecificity. By introducing them into the pathway, the titers of (*R*, *R*)-2,3-BDO significantly increased by 5.8, 5.1, and 6.1 g L^−1^, respectively, from 40 g L^−1^ glucose. All of the enantiomers’ purities could reach 99% [18]. Later, Tong et al. further improved its yield by combining the promoters of different strengths and vectors without the *lac* gene. The purity of the product was higher than 99% and the yield was up to 30.5 g L^−1^ [21]. During the progress of (*S*)-1,2-PDO production, it can be catalyzed from *L*-lactaldehyde by FucO, YqhD or MmsB. To ensure the purity of the product, Zhu et al. tested the specific activity of these three enzymes in vivo, and finally proved that MmsB could efficiently transform the intermediate into (*S*)-1,2-PDO [5].

#### 3.1.4. Protein Engineering of Crucial Enzymes in the Pathways

Moreover, engineering the enzymes themselves could further improve the yield of diols. Yim et al. modified two enzymes in the biosynthetic process of 1,4-BDO. Because the pyruvate dehydrogenase of *E. coli* has an insufficient activity under anoxic or anaerobic conditions, the native gene *lphA* was replaced by *lphA* from *K. pneumonia* [13]. There was also a mutation in the enzyme, which could reduce the sensitivity of the enzyme to NADH [36]. Additionally, R163L was introduced into citrate synthase (encoded by *gltA*), which decreased the inhibition by NADH to enhance the flux of the TCA cycle [13]. For 1,3-PDO biosynthesis, due to the low catalytic activity of wild-type Gdh to *L*-homoserine, Chen et al. established a mutant library of Gdh through silico mutagenesis. By docking screening, Gdh^K92V/T195S^ was obtained that could catalyze *L*-homoserine to 4-hydroxy-2-ketobutyrate with high activity [37]. In addition, in order to decrease the substrate promiscuity of KivD to produce 1,4-BDO, Tai et al. shrank the binding pocket of KivD to reduce the sensitivity to the undesired substrate, and the substrate activity also notably increased [38]. As for 1,4-BDO production from glutamate, in order to improve Glu decarboxylation efficiency, Wang et al. replaced the wild-type GadB with its mutant GadB^E89Q/Δ452−466^. At the same time, the citrate synthase GltA^R163L^ and PPC were overexpressed to increase the carbon flux of glutamate. The 1,4-BDO titer of the host increased by 45.6% [26]. Similarly, Frazão et al. engineered the DHB dehydrogenase and OHB decarboxylase to improve the productivity of 1,3-PDO [39].

Except for deleting the competing pathways or engineering the key enzymes to obtain overproducers, the common method is random mutagenesis for strains such as atmospheric and room-temperature plasma (ARTP) mutagenesis or N-methyl-N′-nitro-N-nitrosoguanidine (NTG)-induced mutagenesis [40]. Therefore, it is necessary to rapidly and effectively screen and select diol overproducers. Until now, biosensors have been widely used in high-yield strains for the screening and regulation of metabolic pathways. By designing, constructing, and modifying the biosensor, it can dynamically respond to the concentration changes of signal molecules. Therefore, the biosensor is a kind of rapid tool that can evaluate mutants with high throughput. BmoR, from *Pseudomonas butanovora*, is an activated transcription factor whose signal molecules are C2–C5 alcohols. When BmoR combines with the signal molecules, it can induce the transcription of the σ^54^-dependent *P_bmo_* promoter [41]. The green fluorescence protein is placed downstream of the *P_bmo_* promoter as a signal representation. The concentration of diols is positively correlated with the amount of GFP expression [42]. Consequently, the fluorescence intensity can be used as an indicator of diol yields. By introducing the biosensor to the mutagenesis library and detecting the GFP expression, the diol overproducers can be efficiently screened.

At the same time, with the rapid development of the artificial design, computer simulation could directly help to analyze the related information about the metabolic network of the chassis by integrating metabolomics, proteomics, and transcriptomics. It could improve the production of desired chemicals, identify the enzymes required in the synthetic process, or even design a pathway that has not been reported. Especially, the biosynthetic pathway of 1,4-BDO was established based on the BNICE algorithm, and the host strain was engineered by the OptKnock algorithm. This computer-simulation tool could help confirm the optimized target in metabolic engineering by the analysis of genome models [13]. Andreozzi et al. analyzed the physiology of 1,4-BDO production in recombined *E. coli* using the kinetic modeling framework ORACLE (Optimization and Risk Analysis of Complex Living Entities) and confirmed the potential strategies to increase the production. Moreover, ORACLE provided the indispensable enzymes that could control 1,4-BDO production and influence the change of enzymes to intracellular states such as cell growth, byproduct formation or redox state [43]. Hence, efficient diol production could be achieved by computer design and analysis.

### 3.2. Optimization of Cofactor Supplementation

Besides the above-mentioned strategies, the optimization of cofactor supplementation is also beneficial for diol production. Numerous NAD(P)H molecules would be consumed during the production process, and the introduction of exogenous pathways or the overexpression of enzymes would also break the balance of NAD(P)H because of the existence of the competing routes. Therefore, sufficient supplementation and the balance of NAD(P)H are essential for stable diol biosynthesis. To solve this problem, numerous studies in past years have balanced the recycling of NAD(P)H by modifying the pathways. Guo et al. maintained the balance of NADPH by coupling 1,3-PDO biosynthesis with the synthetic pathway of methyl butane, since excessive NADPH from methyl-butane production could be utilized to produce 1,3-PDO. Especially, the volatility of methyl butane is beneficial to 1,3-PDO separation and purification after fermentation. With the optimization of the host and fermentation conditions, the yield increased by 3.3–4.3 folds [44]. Liu et al. improved the production of 1,3-BDO by overexpressing *pntAB*, which encodes a membrane-bound proton-translocating transhydrogenase [45]. A novel NADH-regeneration system and a simultaneous acetyl-CoA-regeneration system were established to replenish the consumption in 1,2-PDO production [5]. Besides NAD(P)H, vitamin-B_12_-dependent coenzymes are also required in the biosynthetic process. However, *E. coli* cannot naturally synthesize vitamin B_12_ and its cost is expensive, suggesting the vitamin-B_12_-dependent pathways are not suitable for diol production in industrialization [46,47]. To remove the dependence on vitamin B_12_, Yun et al. produced 1,3-PDO by overexpressing the vitamin-B_12_-independent enzyme GDHt and its activity factor from *Clostridium butyricum* [47]. Li et al. constructed an unnatural pathway of 1,3-PDO derived from acetyl-CoA without supplementation of vitamin B_12_ and the products could be efficiently accumulated [48].

### 3.3. Reprogramming of the Metabolic Network

Furthermore, the engineering of RNA polymerase (RNAP) for reprogramming the metabolic network could further promote the conversion of carbon sources to products. An ideal productive factory should provide maximal resources for production. However, most microorganisms focus on their growth rather than production; the optimization of synthetic pathways cannot fundamentally change the model of resource allocation. As a leader of the gene regulatory system, RNAP controls the proportion of resources allocated to growth and production [49]. Numerous studies have proved that changes to RNAP could adjust the flow of resources to yield its maximum supply production during fermentation. Conrad et al. indicated that the strain containing mutated RpoC had an improved conversion rate of carbon sources compared with the wild type. The conversion rate increased from 15% to 35%, and the growth rate was 1.6-fold higher than that of the reference strain. The speculated reason was that the frequency of transcriptional pausing was reduced, with a consequent increase in the transcript-elongation rate [50]. Guo et al. improved the xylose tolerance and the yield of 2,3-BDO through the direct evolution of RpoD, including changing the amount of *rpoD* expression and its genotype. The yield reached 38.6 g L^−1^ at 62 h, which was increased by 2.3-fold compared with the wild type [51]. At the same time, this strategy has been applied to enhance other compound production [52,53].

The establishment of the mutagenesis library of RNAP is an optionable method to obtain an ideal RNAP mutant. To screen the ideal RNAP mutant, a high-throughput screening method needs to be developed. Nowadays, FACS is widely used in various industries including enzyme modification, strain screening and component optimization [54,55,56]. FACS could be utilized for the screening of RNAP mutagenesis libraries. However, RNAP is a core of the metabolic network, like the global transcription factor. By redesigning the sequence or structure of RNAP, the whole state of the host would be changed. Some introduced mutations might generate negative effects on growth ability, and the mechanism of the mutations of RNAP that influence the metabolic network would be uncertain. When designing the reprogramming strategies, the overall state of the host should be considered in order to improve the production yield without affecting its growth. If the host grows excessively fast, most resources would be used for growth rather than production. The yield of diols would be at a low level. On the contrary, if the host grows slowly, the cell concentration would be limited, resulting that the host could not overproduce diols. Therefore, in order to achieve the continuous production of diols, the allocation of resources between growth and production should be balanced.

## 4. Diol Biosynthesis from Other Carbohydrate Feedstocks

**Figure 2:**
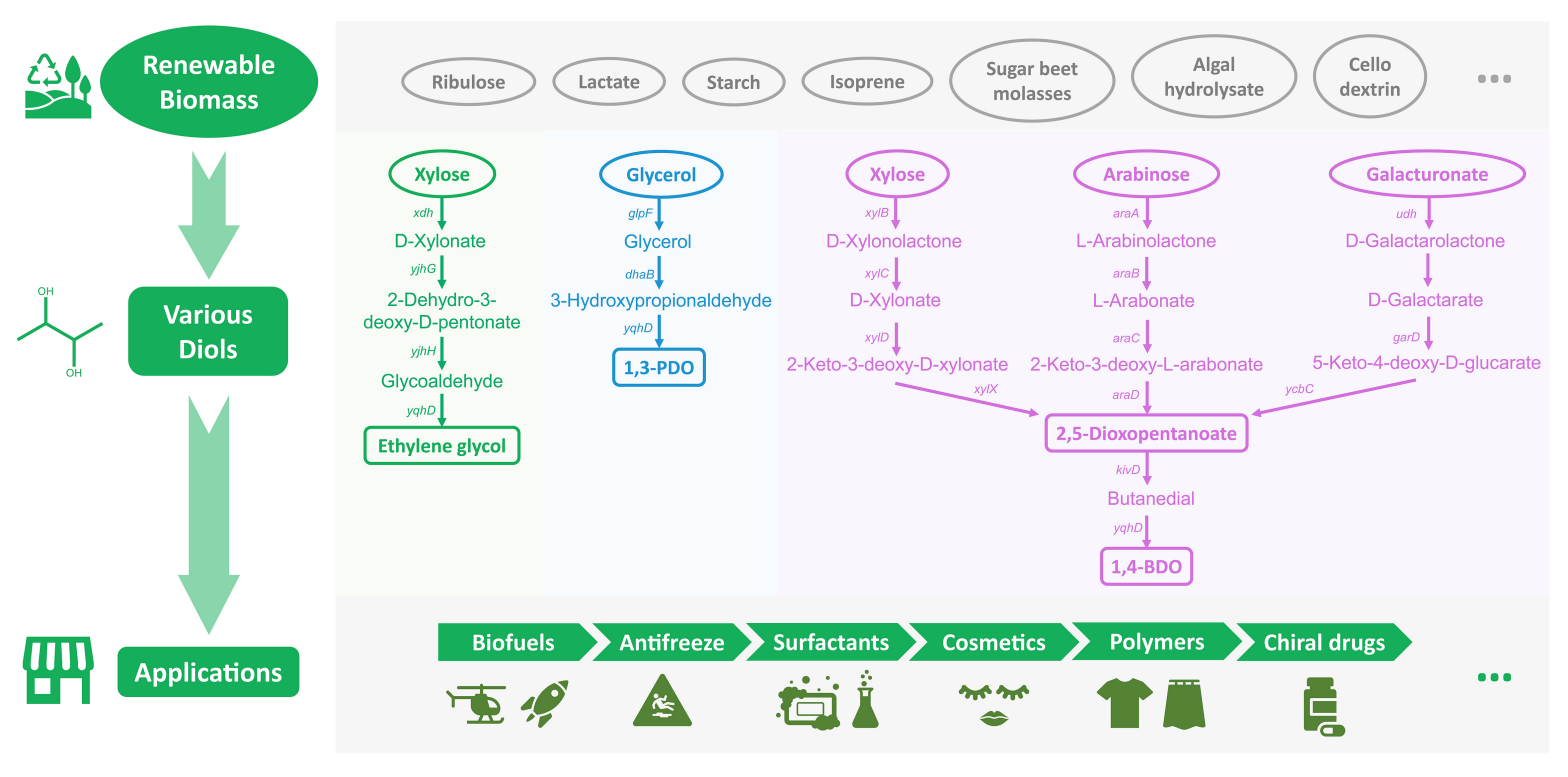
Biosynthetic pathways of diols from other carbon sources in *E. coli*. The green areas represent EG biosynthesis from xylose, the blue areas represent the biosynthetic pathway of 1,3-PDO from glycerol, and the purple areas represent the metabolic pathway of 1,4-BDO from other carbon sources. The gray areas represent the carbon sources for diol biosynthesis, and the applications of diols, respectively. *xdh*: *D*-xylose dehydrogenase; *yjhG*: *D*-xylonate dehydratase; *yjhH*: 2-dehydro-3-deoxy-d-xylonate aldolase; *yqhD*: alcohol dehydrogenase; *glpF*: glycerol facilitator; *dhaB*: glycerol dehydratase; *xylB*: *D*-xylose dehydrogenase; *xylC*: *D*-xylonate dehydratase; *xylD*: 2-dehydro-3-deoxy-d-xylonate dehydratase; *xylX*: α-ketoglutaric semialdehyde dehydrogenase; *araA*: *L*-arabinose dehydrogenase; *araB*: *L*-arabinolactonase; *araC*: *L*-arabonate dehydratase; *araD*: α-keto-3-deoxy-*L*-arabonate dehydratase; *kivD*: α-keto acid decarboxylase; *udh*: uronate dehydrogenase; *garD*: *D*-galactarate dehydratase; *ycbC*: 5-keto-4-deoxy-*D*-glucarate dehydratase.

Except for glucose, other carbon sources such as glycerol, xylose and starch could also be utilized for diol production (Figure 2, Table 2). Because of the pathogenicity, the pathways from glycerol in *Klebsiella* [57,58], *Citrobacter* [59,60], *Clostridium* [61] or *Lactobacillus* [62,63] could not be further used in industrial production, while these well-established routes could be referenced for diol production in *E. coli*. For instance, Tong et al. firstly introduced the 1,3-PDO pathway from *K. pneumoniae* into *E. coil*. After glycerol was transported into the cell, it was gradually dehydrated and oxidized to 1,3-PDO. However, the pathway from glycerol depended on vitamin B_12_, and the accumulated 3-HPA inhibited the activity of GDHt, since the yield of 1,3-PDO was still at a low level [64].

Xylose, a kind of pentose, is one of the raw materials of lignocellulose, in which the content is approximately 20% [81]. With the increase in biomass utilization, diol fermentation from xylose is becoming an absorbing topic [82]. Liu et al. successfully constructed a biosynthetic pathway from *D*-xylose for EG production in *E. coli*. This pathway avoided the high pressure and temperature that are required in the chemical synthesis, and also prevented the side reactions from non-specific carbon–carbon bond cleavages in the hydrogenolysis of xylitol [70]. Subsequently, a de novo biosynthesis of 1,4-BDO from xylose was designed. By engineering PpdA-C-B, the conversion efficiency from 1,2,4-butanetriol to1,4-BDO significantly increased [80].

Various monosaccharides are used to produce diols. However, due to the lack of the ability to assimilate oligosaccharides and polysaccharides, *E. coli* cannot utilize starch as the only carbon source. Recently, the global yield of starch has reached 41 million tons [83]. Reported studies have indicated that chemicals could be produced from starch in *E. coli* such as itaconic acid [84] and polyhydroxybutyrate [85]. Based on this, R. Sato et al. produced 1,2-PDO and 1,3-PDO from starch as the only carbon source for the first time. The diols were successfully biosynthesized by expressing α-amylase from *Streptococcus bovis* NRIC 1535 by the cell-surface display and introducing the reported synthetic enzymes to the host [86].

Compared with fossil fuels, sugar or traditional starch is an environmental-friendly raw material and is convenient for fermentation. However, with the increasing prominence of population and environmental issues, more and more attention has been paid to diol production from industrial and agricultural by-products and waste as the basic raw materials. At the same time, how to efficiently convert diversified wastes into diols has become a research focus in this field. To date, multiple studies have focused on finding alternative sources for diol production, such as using cellobiose in *Saccharomyces cerevisiae* [87], soy hydrolysate in *B. licheniformis* BL1 [88], methane in *Methylotuvimicrobium alcaliphilum* 20Z [89] or agro-industrial residues and cactus cladode acid hydrolysate in *Lactobacillus diolivorans* [90]. Based on this, the utilization of alternative sources in *E*. *coli* is being rapidly propelled. Sathesh-Prabu et al. used the hydrolysate of empty palm-fruit bunches to produce 2,3-BDO by introducing a glucose-induced system from *Pseudomonas putida*, which could be further applied to the production from other lignocelluloses [91]. Erian et al. efficiently utilized sugar-beet molasses to produce 2,3-BDO in pulsed fed-batch cultivations. The productivity was comparable to the productivity from glucose [23]. As a potential biological fuel, algae has high value because of the absence of lignocellulose, which does not need strict pretreatment. Therefore, a study reported that algal hydrolysate was successfully utilized as the carbon resource to synthesize 2,3-BDO and acetoin, and the engineered host obtained a higher 2,3-BDO titer as well [78]. Additionally, it is unignorable that PET has been widely used in various fields of human life such as packaging, agriculture and automobile manufacturing. While convenient, PET also generates many problems. Most plastics including PET are difficult to be degraded and tend to accumulate in the environment, which cause serious environmental damage. It is being widely discussed how to efficiently degrade PET [92]. It has been noted that PET could be converted to EG via gradual degradation [93,94]. Therefore, the degradation products of PET have the potential to become an alternative raw material for diol production in the future.

## 5. Dynamic Regulation of Diol Production

**Figure 3:**
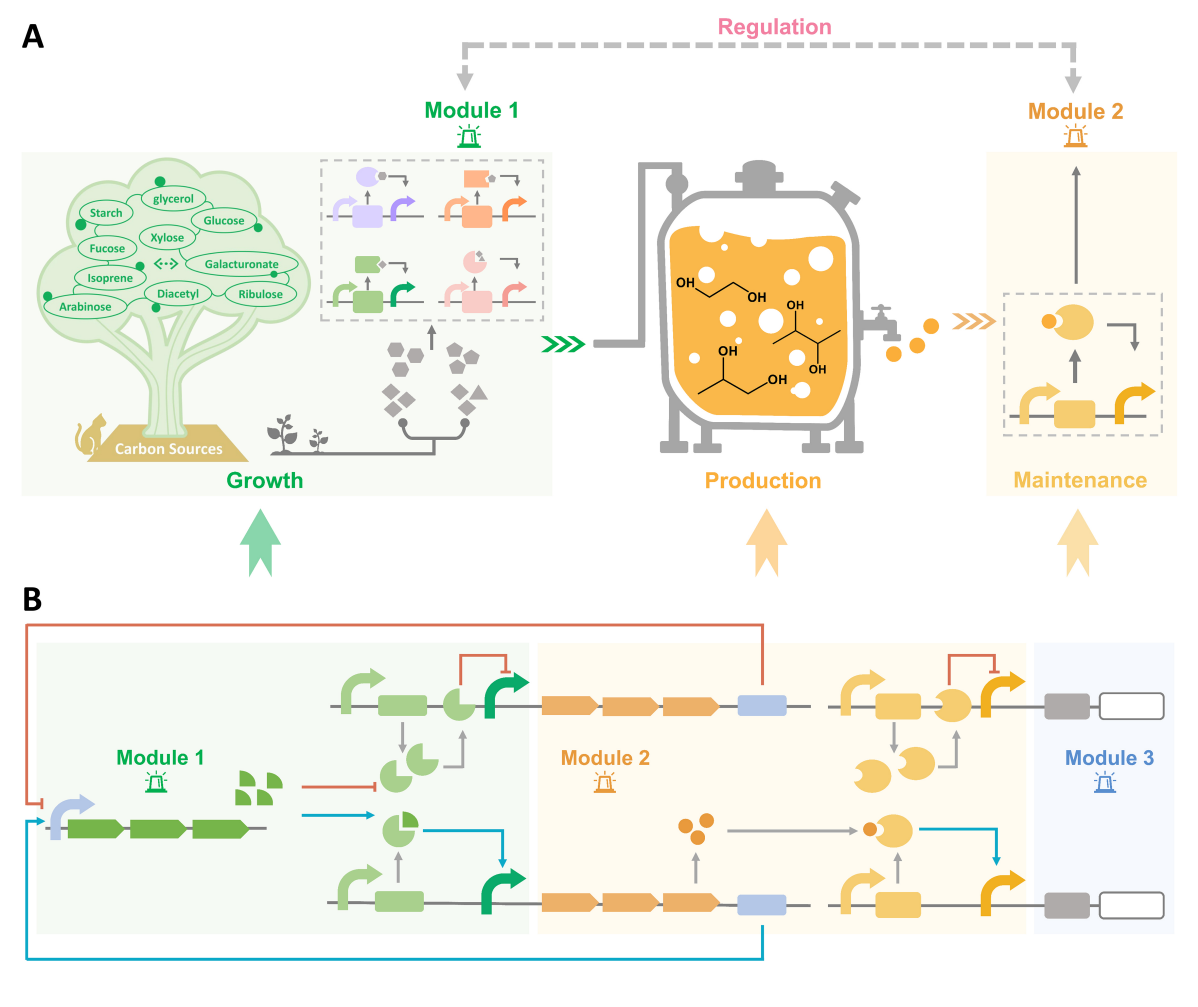
Dynamic regulation of diol production. (**A**) The regulation network between growth and diol production. The growth state and the production state are monitored in real time, and the balance is maintained in the host to achieve the high-level biosynthesis of diols. (**B**) Module design to dynamically regulate diol production. Module 1 detects the supplementation of carbon sources. Module 2 is responsible for the detection of diol yields. Module 3 is designed for controlling the flow of the metabolic flux.

The dynamic regulation of metabolic pathways in microorganisms is an efficient way to maintain the balance of growth and production, which can autonomously regulate gene expression and metabolic flux, as well as detect environmental and intracellular signals. On the contrary, static regulation might lead to an imbalance of the metabolic flux, overaccumulation of intermediates and waste of carbon sources. Nevertheless, in order to improve the productivity, most modifications are designed to direct most carbon sources to production, regardless of growth requirements. As a result, the nutrients for growth would be insufficient, and cell homeostasis would be destroyed. A strategy needs to be established to achieve a dynamic balance between production and growth so that diols can continue to be synthesized with high efficiency. At the same time, the fermentation of chemicals relies on exogenous inducers such as IPTG. However, three factors cannot be ignored: the irreversible induction, the toxicity, and the high cost of inducers. The ultimate purpose of dynamic regulation is to achieve autonomous control of the flux. This tool could be constructed by redesigning promoters or biosensors that respond to metabolites or regulate the transcription of metabolism, or a quorum-sensing system that depends on cell concentrations and group level. Nowadays, dynamic regulation has been applied to produce different compounds in diverse chassis [95,96]. For the regulation of diol production, at least two modules are needed (Figure 3A). The first module is to detect the amount of carbon sources in the cell to satisfy the needs of growth (Module 1). The second module is to control the expression of the key enzymes and monitor the yield of diols (Module 2).

For the monitoring of carbon sources, substrate-induced promoter systems have been established for the regulation of gene expression in *P. putida* KT2440 [97]. The induced chemicals include glucose, xylose, levulinic acid (LA), 3-hydroxypropionic acid (3-HP) and 4-hydroxyvalerate (4-HV), which are the common raw materials or intermediates in the synthetic process. The systems include five categories: (1) the glucose-induced system, HexR-*P_zwf1_* system; (2) the xylose-induced system, XutR-*P_xutA_*; (3) the LA-induced system, LvaR-*P_lvaA_*; (4) the 3-HP/LA-induced system, HpdR-*P_hpdH_*; (5) the 3HP-induced system, MmsR-*P_mmsA_*. The first system has been successfully applied to the production of 2,3-BDO in *E. coli* [91]. As a monitor that could respond to diols, a BmoR-based biosensor could monitor diol production in real time. By combining the two modules to simultaneously regulate growth and production, the host could continuously biosynthesize diols at its best growth state (Figure 3B). In order to better apply the system to regulate production, the modules could be further modified and optimized to have a wider detection range or high sensitivity to substrates and products. In addition, an auxiliary module could be added to control the metabolic flux in order to synthesize other chemicals using tools such as CRISPRi or antisense RNA (Module 3).

## 6. Conclusions and Perspectives

Recently, the biosynthesis of diols has drawn much attention because of its environmental friendliness and high efficiency. The introduction of artificial pathways into *E. coli* could allow for the diol biosynthesis from carbon sources. In addition, a series of metabolic-engineering strategies have been developed and adopted into engineered *E. coli* to enhance diol production, such as the optimization of biosynthetic pathways, the improvement of cofactor supplementation, and reprogramming of the metabolic network. Unfortunately, there are still several limitations for diol production. The first that should be mentioned is that a high concentration of diols would generate toxicity to the chassis. Enhancing the diol tolerance is crucial, such as via the direct evolution of the chassis by continuous culture under selective pressure, or via acceleration of the cell-membrane synthesis in order to increase resistance to diols. The second limitation is the downstream processing of diols. Because of the high boiling point and strong hydrophilicity, the separation and purification of diols are relatively difficult after fermentation [98]. Recent studies indicate that an aqueous two-phase system can be successfully used for the recovery of diols, and the system could be further improved by optimizing extracting solvents to collect different diols [99,100]. In addition, the dependence on cofactors limits diol production, despite various strategies that are designed to optimize the supplementation of cofactors in the synthetic process. Therefore, in order to achieve continuous diol production, the removal of the dependence on cofactors could be a new direction in the future. Nowadays, although transcription-factor (TF)-based biosensors such as BmoR-based biosensor have been widely used to screen high alcohol overproducers, the response of TFs is time-consuming because the response process relies on the transcriptional mechanism. First, the TFs need to combine the effectors to form a complex. Then, the complex would initiate the transcription of the target gene to express a detectable protein, such as GFP. Compared with GC or HPLC, TF-based biosensors could save more time, but they still need a relatively long time to react with effectors. Based on this, TF-based biosensors have the possibility of improvement to achieve nanosecond responses and real-time high-throughput screening. Additionally, studies on using biosensors to screen diol overproducers have not been reported, and the transcription factor with a specific response to diols is absent. Therefore, a de novo design of diol biosensors or the modification of existing biosensors is essential for the detection of diols. With the development of green biology synthesis, diol production from the waste of industrialization or agriculture is rewarded and respected. Biological carbon sequestration is an alternative method to reduce production costs. As a representative of photoautotrophs, cyanobacteria are a superior chassis for producing high-value chemicals without the addition of any exogenous cofactor [101]. For instance, Li et al. engineered a cyanobacterium to achieve 1,2-PDO biosynthesis. Notably, in the production process, 3/4 of the carbon source was from CO_2_ and another 1/4 was from glycogen, which is the storage energy for cyanobacteria [102]. Hence, cyanobacteria have the potential to become a new generation of microbial factory to biosynthesize diols. In addition, the construction of a co-culture system of cyanobacteria and *E. coli* might be an alternative strategy for the production of diols. In this system, cyanobacteria and *E. coli* would be responsible for supplying the carbon source from CO_2_ and for converting the carbon source into diols, respectively. Additionally, *E. coli* has been engineered to generate carbon sources from CO_2_ [103]. In the future, the diol biosynthetic pathways can be introduced into the above-engineered *E. coli* in order to realize diol production from CO_2_ in one host.

## Figures and Tables

**Table 1:** Summary of diols production from glucose in *E. coli*.

Products	Host	Carbon Sources	Knockout/Knock down Genes	Overexpression Genes	Fermentation Conditions	Production (g/L)	Yield (g/g)	Productivity (g/L/h)	Ref.
EG	K-12 MG1655 (DE3)	Glucose	*ΔaldA*, *ΔsdaA*, *ΔeutB*, *ΔeutC*	*serA:317*, *serB*, *serC*, *fucO*, *aao*	Bioreactor	3.1	0.22		[9]
1,2-PDO	AG1	Glucose	None	*gldA*, *mgs*	Shake flask	0.7			[12]
	BW25113	Glucose	*ΔpoxB*, *ΔfrdA*, *ΔmgsA*, *ΔadhE::pdcD*, *ΔlldD::mmsB*, *ΔackA-pta::pct*, *ΔldhA::Lldh*	*pct*, *pdcD*, *mmsB*	Shake flask	1.04			[5]
	AG1	Glucose	*ΔldhA::KanR*	*mgs*, *gldA*, *fucO*	Bioreactor	4.5	0.19		[14]
	BW25113	Glucose	*Δzwf*, *ΔtpiA*, *ΔgloA*, *ΔldhA*, *ΔadhE*,	*mgsA*, *gldA*, *fucO*	Shake flask	5.13	0.48		[15]
1,3-PDO	K-12 MG1655 (DE3)	Glucose	*ΔgldA*, *ΔglpK*, *ΔaldA*, *ΔaldB*, *ΔmgsA*, *ΔptsHI*, replacing *gapA* promoter with the synthetic short 1.5 GI promoter (SEQ ID NO:28)	*galP*	Bioreactor	112	0.26		[15]
	DH5α	Glucose	None	*gpd1-gpp2* fusion gene, *dha operon*, *rpoS*	Bioreactor	12.1			[16]
2,3-BDO	JM109	Glucose	None	*budB*, *budA*, *budC*	Shake flask	*meso-* 17.7	0.27		[17]
	JCL260	Glucose	None	*alsS*, *alsD*, *bdhA from K. pneumoniae*	Shake flask	*(R*, *R)-* 5.8	0.30		[18]
	JCL260	Glucose	None	*alsS*, *alsD*, *adh from C. beijerinckii*	Shake flask	*(R*, *R)-* 5.1	0.29		[18]
	JCL260	Glucose	None	*alsS*, *alsD*, *adh from T. brockii*	Shake flask	*(R*, *R)-* 6.1	0.31		[18]
	BL21(DE3)	Glucose	None	*budB*, *budC*	Shake flask	*(S*, *S)-* 2.2	0.08		[19]
	BL21(DE3)	Glucose	None	*als*, *ar* under control of the constitutive *ackA* promoter	Shake flask	0.66			[20]
	MG1655	Glucose	None	*budA*, *budB*, *ydjL*	Bioreactor	*(R*, *R)-* 30.5	0.38	1.69	[21]
	W023	Glucose	*ΔldhA*,*ΔpflB*, *ΔadhE*, *ΔlpdA::K.p.lpd ^E354 K^*, *Δmdh*, *ΔarcA*	*gltA^R164L^*, *ilvBN*, *aldB*, *bdh1*	Bioreactor	88	0.35	1.87	[22]
	W	Glucose	None	*budA*, *budB*, *budC*	Bioreactor High oxygen	52.1	0.27	4.53	[23]
	W	Glucose	*ΔldhA*, *ΔadhE*, *Δpta*, *ΔfrdA*	*budA*, *budB*, *budC*	Bioreactor Low oxygen	68.1	0.38	1.32	[23]
	JM109	Glucose	*ΔldhA*, *Δpta*, *ΔadhE*, *ΔpoxB*	*alaS*, *alsD*, *budC*	Shake flask	*meso*- 14.5	0.30	0.30	[24]
1,4-BDO	W (ATCC9637)	Glucose	*Δsad::cat2-bld-bdh*, *ΔlacZ::cat1-sucD 4hbd*, *ΔllpdA::K.p.lpdA ^D354K^* *ΔpflB*, *ΔarcA*, *Δmdh*, *ΔadhE*, *ΔldhA*, knock down *tesB*	*gabD*, *ybgC*, *gltA^R163L^*	Bioreactor	1.8			[25]
	K-12 MG1655 (DE3)	Glucose	*ΔadhE*, *ΔldhA*, *ΔpflB*,*Δmdh*,*ΔarcA*, *lpdA::K.p.lpd^D354K^*,	*gltA^R163L^*, *sucA*, *4hbd*, *cat2*, *ald*, *adh*	Bioreactor	18			[13]

**Table 2:** Summary of diols production from other carbon sources in *E. coli*.

Products	Host	Carbon Sources	Knockout/Knock down Genes	Overexpression Genes	Fermentation Conditions	Production (g/L)	Yield (g/g)	Productivity (g/L/h)	Ref.
EG	W3110	Xylose	*ΔptsG*,*ΔlacI*, *P_yqhD_::P_trc_*, knock down *xylC_ccs_*	*xylC_ccs_*, *yqhD*	Bioreactor	108.2	0.36	2.25	[65]
	BL21 (DE3)	Xylose	*ΔarcA*, *ΔaldA*	*xdh*, *xylC*, *yjhG*, *yjhH*, *fucO*	Bioreactor	72.0	0.40	1.38	[66]
	K-12 MG1655 (DE3)	Xylose	*ΔendA*, *ΔrecA*, *ΔxylB*, *ΔaldA*	*dte*, *fucA*, *fucK*, *fucO*	Bioreactor	40.0	0.35		[67]
	K-12 MG1655 (DE3)	*L*-Arabinose	*ΔendA*, *ΔrecA*, *ΔaraB*	*dte*, *rhaB*, *rhaD*, *fucO*	Bioreactor	20.0	0.38		[67]
	MG1655	Xylose	*ΔxylB*, *ΔaldA*	*khkC*, *aldoB*, *fucO*	Shake flask	20.0	0.91	0.37	[68]
	K-12 MG1655 (DE3)	Ribulose	*ΔendA*, *ΔrecA*,*ΔxylB*, *ΔaldA*	*dte*, *fucA*, *fucO*, *fucK*	Shake flask	3.5	0.84	0.35	[69]
	W3110 (DE3)	Xylose	*ΔxylA*	*Xdh*, *yqhD*	Bioreactor	11.7	0.29	0.24	[70]
	W3110 (DE3)	Xylose	*ΔxylAB*, *ΔaldA*, *ΔyjgB*pKMX	*yjgB*, *xdh*	Bioreactor	7.72	0.39		[71]
1,2-PDO	K-12 MG1655 (DE3)	*D*-/*L*-Lactate	*ΔlldD*, *Δdld*,*ΔldhA*, *ΔadhE*	*pct*, *pduP*, *yahK*	Shake flask	(*R*)-1.5 (*S*)-1.7			[72]
	K-12 MG1655 (DE3)	Glycerol	*ΔackA-pta*, *ΔldhA*, *ΔdhaK*	*dhaKL*, *gldA*, *mgsA*, *yqhD*	Bioreactor	5.6	0.21		[73]
1,3-PDO	JM109	Glycerol	None	*dhaB*, *yqhD*, *gdrA*, *gdrB*, *fdh1*, *gapN*, *galP*, *glk*	Shake flask	13.47	0.53		[74]
	K-12 ER2925	Glycerol	None	*dhaB1*, *dhaB2*, *yqhD*	Bioreactor	104.4		2.61	[75]
	Rosetta (DE3) and BL21(DE3)	Glycerol and glucose	None	*dhaB1*, *dhaB2*, *dhaT*	Bioreactor	41.7		0.69	[47]
	BL21(DE3)	Isoprene	*ΔglpK*, *ΔptsG*	*yqhD*, *pntAB*, *galP*, *glk*	Shake flask	2.5			[44]
2,3-BDO	BL21(DE3)	Diacetyl	None	*bdh*, *fdh*	Bioreactor	*(S*, *S)-* 31.7	0.90	2.3	[76]
	JM109	Diacetyl	None	*budC*, *bdh*	Shake flask	*(S*, *S)-* 2.2	0.93		[77]
	W	Sugar beet molasses	None	*budA*, *budB*,*budC*	Fed-batch	56.2	0.44	1.31	[23]
	K-12 MG1655 (DE3)	Algal hydrolysate	*ΔfrdABCD*, *ΔldhA*, *ΔadhE*, *ΔlpdA*, *Δpta*,	*budB*, *budA*, *budC*	Shake flask	*meso*- *(S*, *S)-* 14.1	0.43		[78]
	UT5600	Cellodextrin	*ΔpoxB*, *ΔldhA*, *ΔackA*, *Δpta*,	*alsS*, *alsD*, *budC*, *ced3A*	Shake flask	*meso*- 5.5			[79]
1,4-BDO	BW25113	Xylose	*ΔxylA*, *ΔyagE*, *ΔyjhH*	*xylB*, *xylC*, *xylD*, *yqhD*, *kivD*, *ppdA-C-B^S301A/Q336A/V300M^*	Shake flask	1.51			[80]
	BW25113	*L*-Arabinose	*ΔaraA*, *Δicd*	*araC*, *araD*, *araA*, *araB*, *araE*, *kivd*, *yqhd*	Bioreactor	15.6			[38]
	BW25113	*D*-Galacturonate	*ΔuxaC*, *ΔgarL*, *Δicd*	*udh*, *garD*, *ycbC*, *xylA(CC)*, *kivd*, *yqhd*	Bioreactor	16.5			[38]
	BW25113	Xylose	*ΔxylA*, *ΔyjhH*, *ΔyagE*, *Δicd*	*xylB*, *xylC*, *xylD*, *xylX*, *xylA(CC)*, *kivd^V461I^*, *yqhd*	Bioreactor	12.0			[38]

## Data Availability

Not applicable.
